# Psychological wellbeing of student-athletes: a comparative study between European and American athletes

**DOI:** 10.3389/fpsyg.2025.1604783

**Published:** 2025-07-29

**Authors:** Hugo Keriven, Alberto Sánchez-Sierra, Marta de-la-plaza-San-Frutos, Guillermo García-Pérez-de-Sevilla, Vicente Clemente-Suarez, Eduardo Garcia-Laredo, Marisa Saenz-Bravo, Oluwatoyosi B. A. Owoeye, Diego Miñambres-Martín, Diego Domínguez-Balmaseda

**Affiliations:** ^1^Department of Physiotherapy, Faculty of Medicine, Health and Sports, Universidad Europea de Madrid, Madrid, Spain; ^2^Department of Real Madrid Graduate School, Faculty of Medicine, Health and Sports, Universidad Europea de Madrid, Madrid, Spain; ^3^Faculty of Physiotherapy and Nursing, Universidad de Castilla-La Mancha, Toledo, Spain; ^4^Toledo Physiotherapy Research Group (GIFTO) Toledo, Spain; ^5^Research Group on Exercise Therapy and Functional Rehabilitation, Faculty of Health Sciences, Universidad Europea de Madrid, Madrid, Spain; ^6^Department of Sports Sciences, Faculty of Medicine, Health and Sports, Universidad Europea de Madrid, Madrid, Spain; ^7^Faculty of Health Sciences (PSICOBIOFUN Group), Universidad Internacional de La Rioja (UNIR), Logroño, Spain; ^8^Department of Physical Therapy & Athletic Training, Doisy College of Health Sciences, Saint Louis University, St. Louis, MO, United States; ^9^Masmicrobiota Group, Faculty of Health Sciences, Universidad Europea de Madrid, Madrid, Spain

**Keywords:** injury prevention, stress, recovery, athletes, wellbeing, psychosocial factors

## Abstract

Sports-related injuries occur at rates ranging from 0.5 to 34 per 1,000 practice hours and are a leading cause of early retirement, with psychosocial factors influencing recovery. Injury prevention remains a priority because of its financial and performance impacts on teams. Psychological factors, such as attention disturbances, negative life events, and arousal levels, can impact an athlete’s risk of injury, although psychological interventions are rarely used in sports. Fear of failure, a major stress factor, negatively affects athletes’ motivation and self-perception. Stress and recovery responses vary across cultures, with European and American athletes employing different strategies. Understanding these cultural differences could help tailor interventions to improve performance and wellbeing. This study compared stress and recovery parameters between American and European student-athletes. This observational pilot clinical trial aimed to assess stress and quality of life among young athletes from two universities: Universidad Europea de Madrid UEM and Saint Louis University (SLU), in pre-season conditions. Participants recruited from their respective soccer teams completed a one-time questionnaire to assess social- and sports-related stress. The study followed ethical guidelines and the questionnaires included tools such as the POMS, RESTQ-52, and the Groningen Sleep Questionnaire. This study found significant differences between the groups across various stress categories, including general, emotional, social, and sports-specific stress, as well as fatigue and disturbed breaks, with *p*-values ranging from 0.005 to 0.035. The effect sizes for these differences were moderate, with values ranging from *r* = -0.321 to *r* = -0.429. Overall, significant differences between the groups were observed, with moderate effect sizes for both general and Overall Stress Scores (*r* = -0.397 and *r* = -0.414, respectively). This study highlights significant cultural differences in stress, recovery, sleep quality, and mood between student-athletes at UEM and SLU. These findings emphasize the need for tailored psychological interventions and recovery strategies that address athletes’ unique stressors and cultural environments and improve their performance and wellbeing.

## Introduction

The incidence of sports-related injuries ranges from 0.5 to 34 injuries per 1,000 h of practice, with injuries being a leading cause of early retirement from sports (Junge, 2000). The injury process involves various psychosocial factors that can influence both the duration and quality of an athlete’s return to sports (RTS), potentially delaying or reducing the likelihood of successful comeback. Injured athletes in team sports and sports organizations present both financial and performance-related challenges that affect seasonal outcomes (Wang and Chen, 2024). Therefore, injury prevention remains a key priority for policymakers and sports injury practitioners (Gustafsson et al., 2017; Smith et al., 1990).

Psychological factors are considered intrinsic risk factors that may influence athletes to suffer injuries, making them a necessary point in the injury prevention program (Guo et al., 2025). The present psychological factors contain a series of aspects that can build someone’s personality, such as attention disturbance, arousal levels, daily hassles, and negative life events between others more related to specific tasks dependent on the continuousness that can be worked on by psychological intervention. Psychological interventions have rarely been studied or applied in the sports industry (Gledhill et al., 2018; Zafra et al., 2022).

Fear of failure is a key component of the sports stress process that significantly impacts the elite athletes. Both adolescent and student athletes experience similar levels of stress as professional athletes (Berengüí Gil et al., 2024). Several studies have focused on this aspect, indicating that the fear of failure impacts their negative cognition and concerns regarding the consequences of failure, generating anxiety, worry, fear, stress, and tension. This directly lowers their self-perception and motivation (Grunberg et al., 2024). For the scientific community, several studies in this area highlighting stress as a potential negative outcome for both athletic and everyday life could generate interest in further investigation (Carreira-Míguez et al., 2022; Rodriguez-Besteiro et al., 2022; Sánchez-Conde and Clemente-Suárez, 2021).

A comparison of stress and recovery constructs between European and American athletes is pivotal to understanding the cultural and environmental influences that shape athletes’ psychological responses to competition and training (Stepanyan and Lalayan, 2024). Cross-cultural differences in athletic experiences, including variations in coaching practices, societal expectations, and access to resources, may significantly impact the stress-recovery balance (Graves et al., 2021). For instance, European athletes, particularly those in team-oriented sports, may rely on recovery strategies that vary significantly from their American counterparts, where individualistic performance metrics often dominate (Hardy, 1992). In addition, the psychological and physiological demands placed on athletes across different regions may result in divergent coping mechanisms and recovery practices that influence their overall wellbeing and performance (Fletcher and Hanton, 2001). By exploring these differences, this study sought to examine the broader context in which stress and recovery processes are embedded, contributing to more nuanced interventions tailored to specific cultural and environmental frameworks.

As highlighted previously, it is essential for the scientist community to analyze and understand the stress processes athletes experience during their carriers, to develop new approaches to help them reach peak performance (Gustafsson et al., 2017; Hardy, 1992; Knöbel et al., 2024; Zheng and Ji, 2021).

This study aimed to analyze the potential differences in stress and recovery parameters between American and European student-athletes based on their responses to a psychological questionnaire, highlighting its significance for future research.

## Materials and methods

### Study design

This study was designed as an observational pilot clinical trial specifically targeting young athletes. This study was conducted in accordance with the ethical standards specified in the “World Medical Association Declaration of Helsinki: Ethical Principles for Medical Research Involving Human Subjects” (World Medical Association, 1991). During the study, the CONSORT (Consolidated Standards of Reporting Trials) guidelines were strictly applied to ensure transparency and rigor in the reporting of trial procedures and outcomes.

The study was approved by the Research Ethics Committee under the reference number CIPI/22.095 as proof of its adherence to ethical standards. The study was also registered with the Australian New Zealand Clinical Trials Registry (NCT06855316), as an additional mark of validity for the study’s methodological integrity and ethic (Keriven et al., 2025; Keriven et al., 2023a).

The participants were divided into two distinct groups based on their educational location: the Universidad Europea de Madrid Group (UEM) and the Saint Louis University Group (SLU). Each group participant answered the same questionnaire. The present study was designed to observe potential differences in stress and general QOL between European and American sports students.

Before the start of the study and the collection of the data both in the USA and Spain, an online meeting was conduct with both American and Spanish coaches to obtain details over the session they usually conduct. As the soccer’s training are universal in the world the intensity and time exercise was similar between 1 and 1 h, 20 depending on the day, each session included strength work in the gym, global warm up on the pitch completed by both individual and collective soccer specific task in aim to get ready for the upcoming games. Each session was conducted on the University soccer field both hybrid style (a mixt between an artificial and real grass), as all the session details were homogeneous in both Universities it has been decided to conduct the study.

### Participants

Participants were recruited from the soccer teams of UEM and SLU and selected based on prior agreements and informed consent provided by each player to participate in related studies within which this sub-study was included (Keriven et al., 2023b; Owoeye, 2024; Owoeye et al., 2022, 2024). The study sample consisted of 43 participants with a mean age of 22.74 years, recruited through convenience sampling. Participants were selected based on their affiliation with either the European University of Madrid (*n* = 20) or Saint Louis University (*n* = 23). Recruitment was conducted via direct contact with team coaches and athletic department staff, who facilitated initial communication with players. Interested athletes were then contacted by email and invited to participate voluntarily. Data collection took place during the middle of the competitive season, between regular training and match weeks, avoiding periods immediately before or after major competitions to reduce performance-related variability.

The primary inclusion criterion was active participation in the university’s official soccer team roster during the current season. Athletes were excluded if they were injured or undergoing rehabilitation at the time of data collection, as several items in the assessment tools focused specifically on physical recovery and performance readiness. Although team performance data were not the primary focus of the study, both university teams had comparable competitive records during the season, with more wins than losses, maintaining a stable competitive environment across both groups.

### Procedure

The study’s methodology required participants to complete a one-time assessment, designed through an association of three questionnaires: The Recovery Stress Questionnaire 52 items, The profile of mood states questionnaire and the Groningen sleep questionnaire; to analyze both the social and sports stress related to each university soccer team. Students from both the Saint Louis University and European University of Madrid answered the questionnaires in a current season situation. Each team had to take the questionnaires at the third session of the week when coming at the facilities by the morning. To ensure accuracy and familiarization with the questionnaire complementation, each team was briefed for 20 min before the assessment time. Each time, they did not see the other, as the Spanish resident student completed the assessment within the European University of Madrid (Spain), and those from Saint Louis University performed it within soccer’s university facility department in Saint Louis (Missouri).

### Outcomes

#### Psychological wellbeing

A range of validated psychological questionnaire tools was used to assess the participants’ mental and emotional states within the groups. These include the following:

*The Profile of Mood State (POMS) Questionnaire*: Included 40 items rated on a scale from 0 (“not at all”) to 4 (“Extremely”). Each response was then categorized into one of eight mood state categories to generate a global score. A constant value of 100 was added to each participant to avoid negative values (Petrowski et al., 2021). It has been observed in the literature through several studies that the POMS questionnaire shown a strong value of Chronbach’s alpha internal consistency with a value of 0.80 (Shacham, 1983).

*The Recovery Stress Questionnaire 52 items (RESTQ-52)*: This is a 52-item questionnaire designed to analyze both general and sport-specific recovery and stress. Participants rated each item on a scale from 0 (“Never”) to 6 (“Always”) (Tibbert et al., 2009). As several works were conducted during the decades those showed a good value of Chronbach’s alpha internal consistency between 0.73 and 0.89 (Kellmann and Kallus, 2024; Reynoso-Sánchez et al., 2021).

*The Groningen Sleep Questionnaire*: This questionnaire presents fourteen questions regarding the participants’ sleep experience from the previous night. The participants rated them as 0 (“False”) and 1 (“True”). A higher score indicated a more disturbed sleep (Wingelaar-Jagt et al., 2024). For the present questionnaire the Chronbach’s alpha internal consistency built through several scientific works showed a good value between 0.85 and 0.90 which enhanced its viability (Jafarian et al., 2008; Serrano-Fernández et al., 2021; Simor et al., 2009).

### Statistical analysis

This study included qualitative metrics (Likert scale test) to analyze the significant differences between each group’s subject scores using a non-parametric test, particularly, the Mann-Whitney U test. Prior to conducting inferential analyses, the assumption of normality was assessed using the Kolmogorov-Smirnov test. Based on the results of this normality test, descriptive statistics were reported as either mean and standard deviation for normally distributed data, or median and interquartile range (IQR) for non-normally distributed variables. The effect sizes for significant differences were interpreted using Rosenthal r value. Data analysis was conducted using IBM SPPS Statistics (Version 29.0.2.0, 2023).

## Results

Sociodemographic data of the study population are presented in Table 1. Descriptive statistics for each university are shown in Table 2 (European University of Madrid) and Table 3 (Saint Louis University).

**TABLE 1:** Sociodemographic data of the sample.

Universidad Europea		Saint Louis University	
N	20	N	23
Sex	Men	Sex	Men
Mean of age (years)	24.57	Mean of age (years)	21.15
Mean of height (cm)	171.78	Mean of height (cm)	182.37
Mean of weight (kg)	69.14	Mean of weight (kg)	75.95

**TABLE 2:** Descriptives statistics of the European University of Madrid students.

Index Recovery Stress Questionnaire (RESTQ)			
Minimum	Maximum	Median	IQR
**General stress**			
0.00	5.00	1.925	26.98
**Emotional stress**			
0.000	5.000	2.025	26.98
**Social stress**			
0.50	4.50	2.050	27.75
**Conflicts and pressure**			
0.00	4.50	2.475	26.28
**Fatigue**			
0.00	4.50	2.325	26.60
**Lack of energy**			
1.00	6.00	2.275	25.45
**Somatic complains**			
0.00	4.00	1.700	25.75
**General stress 2**			
0.86	4.36	2.111	27.60
**Success**			
1.00	5.00	3.150	22.23
**Social relaxation**			
0.00	6.00	4.225	24.80
**General wellbeing**			
1.50	6.00	3.800	21.10
**Sleep quality**			
0.50	4.50	2.750	22.83
**General recovery**			
1.70	4.60	3.270	21.08
**Disturbed breaks**			
0.00	3.75	1.737	27.75
**Burn out emotional or exhaustion**			
0.00	3.00	1.437	25.13
**Fitness injury**			
0.00	5.00	2.250	23.38
**Sport specific stress**			
0.58	3.42	1.808	26.45
**Fitness being in shape**			
0.50	5.00	3.262	19.33
**Personal accomplishment**			
1.33	5.00	3.067	18.88
**Self-efficacy**			
1.50	5.75	3.200	19.28
**Self-regulation**			
1.00	5.00	3.225	18.40
**Sport specific recovery**			
1.27	5.19	3.189	18.53
**Overall Stress Score**			
0.22	0.78	0.393	27.48
**Overall Recovery Score**			
0.36	1.09	0.718	19.70
**Groningen Sleep Quality Scale**			
**Minimum**	Maximum	Median	IQR
0	12	4.55	27.30
**Profile of mood states**			
Minimum	Maximum	Median	IQR
80	150	102.50	27.58

**TABLE 3:** Descriptives statistics of the Saint Louis University students.

Index Recovery Stress Questionnaire (RESTQ)			
Minimum	Maximum	Median	IQR
**General stress**			
69.00	116.00	87.478	17.67
**Emotional stress**			
0.00	12.00	2.434	17.93
**Social stress**			
0.00	5.00	0.978	17.00
**Conflicts and pressure**			
0.500	3.000	1.347	18.28
**Fatigue**			
0.00	2.50	1.087	18.00
**Lack of energy**			
0.50	4.00	1.673	19.00
**Somatic complains**			
0.00	4.00	1.500	18.74
**General stress 2**			
0.00	4.00	1.717	17.13
**Success**			
0.00	5.50	1.326	21.80
**Social relaxation**			
0.43	3.00	1.376	19.57
**General wellbeing**			
1.50	5.50	4.043	21.10
**Sleep quality**			
0.50	5.00	2.934	22.83
**General recovery**			
1.50	5.50	4.000	22.80
**Disturbed breaks**			
0.00	5.00	2.608	17.00
**Burn out emotional or exhaustion**			
1.10	4.40	3.356	19.28
**Fitness injury**			
0.00	3.50	0.923	20.80
**Sport specific stress**			
0.00	3.50	1.065	18.13
**Fitness being in shape**			
0.00	4.75	2.000	24.33
**Personal accomplishment**			
0.08	3.42	1.330	24.72
**Self-efficacy**			
2.25	5.50	3.771	24.37
**Self-regulation**			
2.33	5.67	3.608	25.13
**Sport specific recovery**			
1.25	6.00	3.728	25.02
**Overall Stress Score**			
2.00	5.25	3.804	17.24
**Overall Recovery Score**			
2.33	5.42	3.729	24.00
**Groningen Sleep Quality Scale**			
Minimum	Maximum	Median	IQR
0	12	2.43	17.39
**Profile of mood states**			
Minimum	Maximum	Median	IQR
69	116	87.48	17.15

There is a constant significant difference between groups, as shown in Table 4, for some categories linked with stress, specifically in the following: General Stress (*p* = 0.013); Emotional Stress (*p* = 0.021); Social Stress (*p* = 0.005); Conflicts and Pressure (*p* = 0.035); Fatigue (*p* = 0.024); General Stress 2 (*p* = 0.006): Disturbed Breaks (*p* = 0.005); Sport Specific Stress (*p* = 0.03); Overall Stress Score (*p* = 0.008).

**TABLE 4:** U of Mann-Whitney results for independents sample between the groups for the Index Recovery Stress Questionnaire (RESTQ) test.

Item RESTQ	U of Mann-Whitney	*Z*	*p*-value	R of Rosenthal
General stress	130.500	−2.475	0.013	−0.377
Emotional stress	136.500	−2.316	0.021	−0.353
Social stress	115.000	−2.831	0.005	−0.431
Conflicts and pressure	144.500	−2.107	0.035	−0.321
Fatigue	138.000	−2.261	0.024	−0.344
Lack of energy	161.000	−1.705	0.088	–
Somatic complains	155.000	−1.854	0.064	–
General stress 2	118.000	−2.731	0.006	−0.416
Success	225.500	−0.111	0.912	–
Social relaxation	174.000	−1.378	0.168	–
Somatic relaxation	158.500	−1.766	0.077	–
General wellbeing	212.000	−0.442	0.659	–
Sleep quality	213.500	−0.412	0.680	–
General recovery	211.500	−0.452	0.652	–
Disturbed breaks	115.000	−2.819	0.005	−0.429
Burn out emotional or exhaustion	167.500	−1.532	0.126	–
Fitness injury	202.500	−0.672	0.502	–
Sport specific stress	141.000	−2.170	0.030	−0.33
Fitness being in shape	176.500	−1.309	0.191	–
Personal accomplishment	167.500	−1.532	0.126	–
Self-efficacy	175.500	−1.331	0.183	–
Self-regulation	158.000	−1.758	0.079	–
Sport specific recovery	160.500	−1.693	0.090	–
Overall Stress Score	120.500	−2.669	0.008	−0.407
Overall Recovery Score	184.000	−1.121	0.262	–

The effect size results indicated by Rosenthal’s r value for the previous items showed a moderate effect size for: General Stress (*r* = -0.377); Emotional Stress (*r* = -0.353); Social Stress (*r* = -0.431); Conflicts and Pressure (*r* = -0.321); Fatigue (*r* = -0.344); General Stress 2 (*r* = -0.416), Disturbed Breaks (*r* = -0.429); Sport Specific Stress (*r* = -0.33) and Overall Stress Score (*r* = -0.407).

A significant difference was observed between the groups (*p* = 0.009) (Table 5) with a moderate effect size (*r* = -0.397) as indicated by Rosenthal’s *r* value.

**TABLE 5:** U of Mann-Whitney results for independents sample between the groups for Groningen Sleep Quality Scale and the Profile of Mood States (POMS) test.

Test	U of Mann-Whitney	Z	*p*-value	R of Rosenthal
Groningen Score	124.000	−2.605	0.009	−0.397
POMS Score	118.500	−2.716	0.007	−0.414

A significant difference was observed between the groups (*p* = 0.007) (Table 5) with a moderate effect size (*r* = -0.414) as indicated by Rosenthal *r* value.

## Discussion

This study provides significant insights into the stress and recovery dynamics of student-athletes from two distinct cultural and academic backgrounds: Universidad Europea de Madrid (UEM) and Saint Louis University (SLU). Using validated psychological tools, we identified meaningful differences in stress and recovery indicators between the two groups, which suggests that stressors in sports environments may be influenced by both cultural and environmental factors. These findings contribute to the existing literature on sports psychology, particularly regarding how cultural contexts affect stress, recovery, and overall athletic wellbeing (Martínez-Pascual et al., 2022; Martín-Rodríguez et al., 2021).

### Stress differences and the literature

Significant differences were found between groups across several categories of stress, including general stress, emotional stress, social stress, conflict and pressure, and fatigue. European athletes from UEM reported higher levels of these stressors, with the *General Stress* category showing the most notable difference (*p* = 0.013), which is supported by existing research highlighting how stress can vary across different cultural contexts (Smith et al., 1990). Similar results were reported by Gustafsson et al. (2017) who observed that athletes in high-pressure environments often face heightened stress levels that can influence their recovery and performance outcomes (Gustafsson et al., 2017).

This aligns with studies suggesting that athletes from collectivist cultures, such as those in European countries, may experience more interpersonal stress owing to societal expectations, social relationships, and pressure to perform for the team (Oyserman et al., 2002). In contrast, American athletes at SLU reported lower stress in several areas, including Emotional Stress (*p* = 0.021) and Sport-Specific Stress (*p* = 0.030), potentially indicating that athletes in more individualistic cultures may have varying coping mechanisms or more personal space to manage these stressors. This finding mirrors that of Lee et al. (2023) who examined the role of cultural differences in how athletes perceive and react to stress, suggesting that athletes from more individualistic cultures exhibit more autonomous coping strategies, which might help explain their lower stress levels (Lee et al., 2023).

However, some stress-related variables did not show statistically significant differences between groups, which was unexpected. One possible explanation is the presence of moderating variables such as individual personality traits, previous exposure to competitive environments, or varying levels of social support from peers and coaches. These factors might buffer the effects of cultural stressors in certain individuals. Additionally, differences in stress perception could also be influenced by sport-specific demands, which were not equally distributed across groups, and this could have introduced variability. Future studies should consider these potential moderators to better understand under what conditions cultural differences exert their greatest influence on athlete stress responses.

### Sleep quality and mood states

In addition to stress, significant differences in sleep quality and mood were observed between the two groups. The Groningen Sleep Quality Scale (*p* = 0.009) and Profile of Mood States (POMS) (*p* = 0.007) both indicated that athletes at SLU experienced better sleep quality and more positive mood states than their UEM counterparts. These findings are consistent with those of Gledhill et al. (2018), who emphasized the critical role of sleep quality in athlete recovery, and Carreira-Míguez et al. (2022), who linked sleep disturbances and negative mood states to increased injury risk and decreased performance. Better sleep and mood in American athletes may be linked to different lifestyle factors, such as more structured schedules, better sleep hygiene education, or a potentially less intense academic and athletic environment compared to the European context (Carreira-Míguez et al., 2022; Gledhill et al., 2018).

Nevertheless, the absence of stronger differences in some mood-related subscales also warrants discussion. It is plausible that mood states are more susceptible to short-term fluctuations and contextual influences, such as competition schedules or academic stress during data collection, which might have diluted group-level differences. Moreover, individual-level variables such as resilience, emotional regulation strategies, and prior mental health status could have moderated the effects observed, masking potential group effects.

### Implications for practice

The significant differences between the groups highlight the need for tailored psychological interventions and recovery strategies based on athletes’ unique stressors and cultural environments. Gustafsson et al. (2017) argued that psychological interventions targeting stress management could significantly improve athletes’ recovery and overall wellbeing. This aligns with our findings, suggesting that interventions focusing on emotional and social stress management could be particularly beneficial for European student-athletes who experience higher levels of these stressors. Moreover, promoting better sleep hygiene and mood regulation, particularly in athletes experiencing high stress levels, may be associated with improved recovery outcomes and performance (Sánchez-Conde and Clemente-Suárez, 2021).

### Limitations and future lines of research

Although this study provides significant insights into the role of culture and environment in stress and recovery, further research is required to explore these differences in a broader sample of athletes across various sports and academic institutions, and in female athletes. Studies should investigate the underlying mechanisms that contribute to these differences, including how social support, coaching styles, and institutional factors impact stress and recovery in athletes from varying cultural backgrounds. Future research should examine the longitudinal effects of these stress factors on athletic performance, injury rates, and long-term wellbeing. Given the cross-sectional nature of our design, we emphasize that our findings reflect associations rather than causal relationships. Thus, causal interpretations should be avoided. As a limitation of the present study, we should mention the sample size knowing that a conventional sample was used, it could be relevant to increase that number and diversify the sports implicated in a similar study in the future.

## Conclusion

This study highlights the significance of considering cultural and environmental factors when designing sports psychology interventions. By acknowledging these differences, sports psychologists, coaches, and healthcare professionals can tailor their approaches to better support the mental and physical wellbeing of athletes from diverse backgrounds. Our findings suggest that American and European student-athletes differ in key stress and recovery indicators, reinforcing the importance of culturally sensitive assessment and intervention strategies.

Given the cross-sectional nature of the study, these results should be interpreted as associations rather than causal relationships. Nevertheless, they provide a valuable foundation for future investigations into how cultural context may shape athletes’ psychological responses and recovery processes. Expanding this line of research with larger, more diverse samples and longitudinal designs will help clarify the role of individual and institutional factors in moderating these effects.

## Data Availability

The raw data supporting the conclusions of this article will be made available by the author, without undue reservation.
